# Pharmaceutical-industry R&D and hospital financial performance in Japan: a fixed-effects analysis

**DOI:** 10.1016/j.hpopen.2026.100181

**Published:** 2026-08-20

**Authors:** Yuto Yoshinaga, Takahiro Inoue

**Affiliations:** a Department of Accountancy (Accounting School), Graduate School of Economics and Management, Tohoku University, 2-1-1 Katahira, Aoba-ku, Sendai, Miyagi, Japan; b Healthcare Management Research Center, Chiba University Hospital, Chiba, Chiba, Japan

**Keywords:** R&D, Pharmaceutical cost, Hospital, Financial performance, Fixed effects, Japan

## Abstract

**Background:**

Rising pharmaceutical prices and increasing research and development (R&D) expenditure by pharmaceutical companies have raised concerns about hospital financial sustainability worldwide. Nevertheless, evidence on the association between pharmaceutical-industry R&D expenditure and hospital financial performance remains limited.

**Objectives:**

To examine whether the associations between pharmaceutical-industry R&D expenditure and hospital financial performance differ between the special functioning hospital category, which provides advanced medical care, and other hospital categories.

**Methods:**

This study integrated annual financial data for four institutionally defined hospital categories with firm-level R&D data for listed pharmaceutical companies. Hospital data were obtained from a national survey by Japan's Ministry of Health, Labour and Welfare. Firm-level R&D data from NEEDS-Financial QUEST 2.0, eol, and company disclosures were aggregated into an industry-level measure aligned with the hospitals' fiscal year. Hospital-category fixed-effects models were used in the analyses.

**Results:**

The association between pharmaceutical-industry R&D expenditure and the pharmaceutical cost ratio was more positive for the special functioning hospital category than for the other categories. The association with the medical operating margin was also more negative for this category.

**Conclusions:**

Higher R&D expenditure was associated with less favorable financial indicators for the special functioning hospital category than for the other categories. These findings suggest that hospitals providing advanced medical care may face greater financial pressures related to pharmaceutical R&D and high-cost medicines, although hospital-level evidence is needed to confirm this interpretation.

## Highlights

• Pharmaceutical-industry R&D and hospital financial indicators were examined.
• Pharmaceutical cost-ratio links were more positive for special functioning hospitals.
• The profitability association was more negative for special functioning hospitals.
• Hospitals providing advanced medical care may face greater financial pressures.
• Hospital-level evidence is needed before targeted support can be considered.

## 1 Background

Rising pharmaceutical expenditure has generated growing concern about the financial sustainability of healthcare systems worldwide. A recent multi-country European report documented steady increases in pharmaceutical expenditure and identified higher prices, particularly for novel medicines, as a major driver of this growth [1]. In the United States, President Donald Trump signed an executive order in 2025 aimed at bringing the prices paid by Americans and taxpayers for prescription drugs closer to those paid in comparable countries [2]. In Japan, the National University Hospital Directors' Conference projected further deterioration in the financial performance of national university hospitals because of rising operating costs and noted that pharmaceutical costs were also expected to increase [3].

The discovery and development of new pharmaceuticals require substantial research and development (R&D) investment and involve long development periods, complex preclinical and clinical research, and a high risk of failure [4], [5], [6], [7], [8]. R&D expenditure represents an input into this process and may be associated with the development of innovative medicines, although greater expenditure does not necessarily lead to a proportional increase in successful products [9], [10]. However, the relationship between pharmaceutical-industry R&D expenditure and drug prices does not reflect a simple and direct cost pass-through [8], [11]. The revenues generated by successfully commercialized products may therefore contribute to recovering the costs of both successful and unsuccessful development projects [4], [5]. The extent to which development costs are reflected in drug prices nevertheless depends on the institutional rules governing pharmaceutical pricing.

This institutional qualification is particularly important in Japan, where reimbursement prices are administratively determined rather than freely established by pharmaceutical firms [12]. Japan uses two principal approaches to determine the prices of new drugs: the comparative method and the cost calculation method [12], [13]. When an appropriate comparable drug exists, the price is determined under the comparative method, primarily with reference to the daily price of the most similar drug [12], [13]. Adjustments may be applied for innovativeness, clinical usefulness, marketability, pediatric indications, and expedited development [12], [13]. Under this method, pharmaceutical firms' R&D expenditure is not directly incorporated as a product-specific cost, although product characteristics associated with innovation and clinical value may be reflected through applicable premiums [12]. When no appropriate comparable drug exists, the cost calculation method is applied [12]. Under this method, the price is calculated by adding selling, general, and administrative expenses, operating profit, distribution costs, and taxes to manufacturing costs [12]. R&D expenditure may therefore be reflected as part of selling, general, and administrative expenses.

These pricing rules indicate that higher firm-level R&D expenditure does not mechanically determine the price of every new drug. However, some newly developed pharmaceuticals may receive relatively high reimbursement prices based on their innovativeness or clinical usefulness. After listing, reimbursement prices are periodically revised primarily on the basis of surveyed market prices and may also be subject to market-expansion repricing and cost-effectiveness-based adjustments [12]. Hospitals that use newly developed and high-cost pharmaceuticals more intensively may consequently incur greater pharmaceutical expenditure.

Japan provides a relevant setting for this study because its rapidly aging population has intensified concerns about rising medical expenditure. Medical service fees are regulated and all citizens are covered by public health insurance, which guarantees access to affordable and advanced medical care [14]. Since 2000, the overall revision rate after accounting for drug price revisions has generally been negative [15]. Consequently, many public hospitals in Japan have difficulty covering their medical expenses with their medical revenue [16].

As part of healthcare reform, Japan introduced the Diagnosis Procedure Combination/Per-Diem Payment System (DPC/PDPS) in 2003 [17], [18]. By June 2024, DPC/PDPS hospitals were expected to account for approximately 85% of beds classified under acute-care general hospitalization fees [19]. The DPC/PDPS is similar to the diagnosis-related group (DRG)-based prospective payment system used in the US Medicare program but differs in providing per-diem rather than per-case reimbursement [17]. Under DPC/PDPS, pharmaceuticals administered during inpatient care are generally included in bundled payments [17], [19]. However, some new and expensive drugs are reimbursed separately on a fee-for-service basis [18], [19]. Therefore, greater use of expensive pharmaceuticals may be associated with a higher pharmaceutical cost ratio and a lower medical operating margin when the corresponding increase in acquisition costs is not fully offset by reimbursement.

Not all hospital categories may be equally exposed to the financial pressures associated with newly developed and high-cost pharmaceuticals. As of October 1, 2024, 88 hospitals in Japan were designated as special functioning hospitals [20]. These hospitals are institutionally responsible for providing advanced medical care, developing and evaluating advanced medical technologies, and training healthcare professionals. Given these responsibilities, special functioning hospitals may be more likely than hospitals in the other categories examined in this study to use newly developed and high-cost pharmaceuticals.

Despite the potential relevance of this mechanism, prior studies have not examined whether the associations between pharmaceutical-industry R&D expenditure and hospital financial performance differ across hospital categories. This research question may also have broader relevance because Japan shares institutional features with other publicly financed healthcare systems. In several high-income countries, public authorities or payers negotiate reimbursement prices for brand-name drugs with manufacturers [21]. DRG-based prospective payment is also widely used for acute inpatient care [22].

To address this gap, this study examines whether these associations are more pronounced for the special functioning hospital category than for the other hospital categories. Because special functioning hospitals may use innovative pharmaceuticals more intensively, the association between pharmaceutical-industry R&D expenditure and the pharmaceutical cost ratio should be more positive for this category. Furthermore, because hospitals have limited ability to offset increases in pharmaceutical acquisition costs under Japan's regulated reimbursement system, the association between pharmaceutical-industry R&D expenditure and the medical operating margin should be more negative for this category. Accordingly, the following hypotheses were developed:

H1: The association between pharmaceutical-industry R&D expenditure and the pharmaceutical cost ratio is more positive for the special functioning hospital category than for the other hospital categories.

H2: The association between pharmaceutical-industry R&D expenditure and the medical operating margin is more negative for the special functioning hospital category than for the other hospital categories.

## 2 Methods

### 2.1 Models and variables

The dataset used in this study is available as Supplementary material 1 in Appendix A. This study did not involve human participants, animals, or hazardous materials. Therefore, ethical approval and informed consent were not required. The statistical analyses in this study were performed using StataNow MP version 19.5.

Eqs. (1), (2) were used to test H1 and H2, respectively. The subscripts *i* and *t* represent hospital categories and years, respectively. The primary models were estimated using the “areg” command in Stata, absorbing hospital-category fixed effects and applying year-cluster robust standard errors. Because the sample contained only 14 fiscal-year clusters, conventional cluster-robust inference may be less reliable. This study therefore supplemented the inference with wild cluster bootstrap tests for the coefficient on *R&D × SFH*. The tests used 9,999 replications, Rademacher weights, and clustering by fiscal year [23].

(1) $${\mathrm{PCR}}_{i,t}=\alpha +\beta_{1}R\&D_{t}+\beta_{2}R\&D_{t}\times {\mathrm{SFH}}_{i}+\beta_{3}{\mathrm{Trend}}_{t}+\delta_{i}+\varepsilon_{i,t}$$

(2) $${\mathrm{Prof}}_{i,t}=\alpha +\beta_{1}R\&D_{t}+\beta_{2}R\&D_{t}\times {\mathrm{SFH}}_{i}+\beta_{3}{\mathrm{Trend}}_{t}+\delta_{i}+\varepsilon_{i,t}$$

The pharmaceutical cost ratio (*PCR*) and medical operating margin (*Prof*) were calculated annually for each of the four hospital categories. *PCR* was calculated by dividing pharmaceutical costs by medical revenue. *Prof* was calculated by dividing the medical operating balance by medical revenue. *R&D* was measured as the natural logarithm of the average R&D expenditure of the sampled pharmaceutical firms. *SFH* was defined as an indicator equal to one for the special functioning hospital category and zero for the other three hospital categories. Additionally, a linear trend variable (*Trend*) was included as a parsimonious control for common time trends in the dependent variables.

The primary models included hospital-category fixed effects (*δᵢ*), which control for time-invariant differences across the four hospital categories and estimate the associations using within-category variation over time. Because the analysis concerned four specific hospital categories rather than a random sample of categories, fixed effects were adopted without imposing the random-effects assumption that unobserved category-specific effects are uncorrelated with the explanatory variables. As a supplementary statistical assessment, conventional Hausman tests based on model-based covariance estimates were conducted. The null hypothesis of no systematic difference between the fixed-effects and random-effects estimators was rejected for the *PCR* model but not for the *Prof* model. The results were interpreted cautiously because the panel contained only four hospital categories and the tests produced rank-deficiency warnings. Given the substantive rationale for controlling for time-invariant differences across the four categories, together with the supplementary evidence from the *PCR* model, the fixed-effects specification was applied consistently to both outcomes to maintain comparability.

The standalone *SFH* indicator was not included in Eqs. (1), (2) because it is time invariant within each hospital category. In the dataset, *SFH* equals one for the special functioning hospital category and zero for the other three categories throughout the study period. The *SFH* indicator is therefore perfectly collinear with the hospital-category fixed effects and is fully absorbed by them. Consequently, its standalone coefficient cannot be separately estimated.

In this specification, *β*_1_, the coefficient on *R&D*, represents the association between pharmaceutical-industry R&D expenditure and the outcome for the three non-SFH categories. The coefficient on *R&D × SFH*, *β*_2_, represents the difference in this association between the special functioning hospital category and the three non-SFH categories.

H1 was considered supported if *β*_2_ in Eq. (1) was significantly positive, indicating that the association between pharmaceutical-industry R&D expenditure and the pharmaceutical cost ratio was more positive for the special functioning hospital category than for the other three non-SFH categories. H2 was considered supported if *β*_2_ in Eq. (2) was significantly negative, indicating that the association between pharmaceutical-industry R&D expenditure and the medical operating margin was more negative for the special functioning hospital category than for the three non-SFH categories.

To assess the sensitivity of the results to the specification of the common time pattern, this study conducted two additional analyses. First, a quadratic time trend (*Trend*^*2*^) was added to each primary model while retaining the linear time trend. This specification allowed the outcomes to follow nonlinear paths over the study period. Second, the common linear time trend was replaced with a full set of year fixed effects to flexibly account for nonlinear changes, year-specific shocks, and potential structural changes associated with policy revisions. Because *R&D*, *Trend*, and *Trend*^*2*^ vary only across years, their standalone coefficients are perfectly collinear with the year fixed effects and cannot be separately estimated in the year fixed-effects specification. However, the interaction term *R&D × SFH* remains identifiable because it varies across both years and hospital categories. The specification with year fixed effects therefore provides an additional robustness test of H1 and H2.

### 2.2 Hospital-category financial data

The unit of analysis was the hospital category-year. Each observation therefore represented the aggregated financial data for a hospital category in a given fiscal year. Annual hospital-category financial data were obtained from the Ministry of Health, Labour and Welfare's “Survey on the Actual State of Medical Care Economy (Survey of Medical Institutions, etc.)” [24]. In this survey, aggregated financial data are separately reported for four institutionally defined categories: special functioning hospitals, dental university hospitals, DPC hospitals, and children's hospitals. Detailed definitions and the treatment of overlap among the four categories are provided in Table 1. The use of these categories is institutionally grounded because they correspond to categories separately reported in the source survey rather than classifications constructed by the authors. Medical revenue, pharmaceutical costs, and the medical operating balance were extracted to calculate *PCR* and *Prof*.

**Table 1:** Definitions and institutional basis of the four hospital categories

Hospital category	Definition and institutional basis
Special functioning hospitals	Special functioning hospitals are hospitals approved by the Minister of Health, Labour and Welfare under Article 4–2 of Japan's Medical Care Act. They must have the capacity to provide advanced medical care, develop and evaluate advanced medical technologies, provide training in advanced medical care, and ensure a high level of medical safety, in addition to satisfying other statutory requirements.
Dental university hospitals	Dental university hospitals are medical institutions affiliated with university schools or graduate schools of dentistry.
DPC hospitals	DPC hospitals are hospitals participating in the DPC/PDPS.
Children's hospitals	Children's hospitals are facilities recognized by the Japanese Association of Children's Hospitals and Related Institutions as having been established and operated to provide advanced and comprehensive medical care for children and adolescents.

Notes: The table summarizes the definitions and institutional basis of the four hospital categories reported in the source survey. Hospitals participating in the DPC/PDPS that are assigned to one of the other categories are excluded from the DPC hospital category. Special functioning hospitals are also excluded from the children's hospital category. The categories are therefore reported without overlap in the source survey.

### 2.3 Pharmaceutical-industry R&D data

Quarterly R&D data for listed companies were obtained mainly from NEEDS-Financial QUEST 2.0 [25], which contains financial data for listed companies in Japan. The study focused on companies classified as “pharmaceuticals” under the Tokyo Stock Exchange Industry Classification. Eligible companies had fiscal years ending in March, June, September, or December and available R&D data. Data were obtained in the following order of priority: “[IFRS] Research and Development Expenses [Cumulative Total]” (FINFSTA'D01148), “Development Expenses and Experiment and Research Expenses” (FINFSTA'K01082), and “Research and Development Expenses [Cumulative Total]” (FINFSTA'H01033). When all three items were missing, R&D data were manually collected from disclosure materials available on the relevant company websites or through eol [26]. Details of the R&D data sources of the sample firms and the manual data-collection procedures are provided as Supplementary material 2 in Appendix A.

Hospital financial data were annual, whereas pharmaceutical firms' R&D data were quarterly. The fiscal year of most Japanese hospitals ends in March, whereas the fiscal-year-end month varies by pharmaceutical firm. To align firm-level R&D data with the hospital fiscal year, quarterly R&D data were aggregated over the 12-month period from April to the following March. For firms with fiscal years ending in March, June, September, or December, the relevant disclosures were used to derive R&D expenditure for each calendar quarter. The amounts for April–June, July–September, October–December, and the following January–March were then summed to obtain annual R&D expenditure for each hospital fiscal year. After the above processing, firms without complete quarterly data for the entire sample period were excluded to maintain a constant firm composition in the industry-level R&D measure. Biofermin Pharmaceutical was also excluded to avoid double counting because it became a consolidated subsidiary of Taisho Pharmaceutical (Holdings) in March 2008. Finally, the industry-level R&D measure was calculated using data for the remaining 42 companies listed in Supplementary material 2 in Appendix A.

## 3 Results

Table 2 presents the descriptive statistics. Panel A reports statistics for the full sample, whereas Panel B reports the means and standard deviations of *PCR* and *Prof* by hospital category. The mean *PCR* differed substantially across the four categories, ranging from 0.059 for dental university hospitals to 0.243 for special functioning hospitals. The mean *Prof* also varied considerably, ranging from −0.559 for dental university hospitals to −0.035 for DPC hospitals. These descriptive differences underscore the importance of accounting for hospital-category-specific heterogeneity in the regression analyses. This sample comprised 56 observations across four hospital categories from the fiscal years 2009 to 2022. The maximum value of *Prof* was negative, indicating that the medical operating balance was negative in every category-year observation in the sample. This is consistent with a previous study reporting that 72.8% of Japanese hospitals had medical losses [16].

**Table 2:** Descriptive statistics.

Panel A. Full sample								
	Mean	SD	Min	25%	Median	75%	Max	N
*PCR*	0.155	0.068	0.039	0.115	0.153	0.207	0.280	56
*Prof*	−0.199	0.220	−0.779	−0.294	−0.095	−0.057	−0.001	56
*R&D*	10.627	0.165	10.430	10.475	10.580	10.736	11.028	56
*SFH*	0.250	0.437	0.000	0.000	0.000	0.500	1.000	56
*Trend*	6.500	4.068	0.000	3.000	6.500	10.000	13.000	56

Panel B. Descriptive statistics of *PCR* and *Prof* by hospital category					

	Mean *PCR*	SD of *PCR*	Mean *Prof*	SD of *Prof*	N
SFH	0.243	0.023	−0.072	0.017	14
DUH	0.059	0.020	−0.559	0.109	14
DPC	0.148	0.004	−0.035	0.023	14
CH	0.170	0.017	−0.131	0.038	14

Notes: Panel A presents descriptive statistics for the full sample. Panel B presents the means and standard deviations of *PCR* and *Prof*. SFH, DUH, DPC, and CH represent special functioning hospitals, dental university hospitals, DPC hospitals, and children's hospitals, respectively.

Table 3, Table 4 present the results from regressing *PCR* and *Prof* on the explanatory variables, respectively. Column (2) reports the primary specification for Eqs. (1), (2), whereas Column (1) includes only *R&D* and *R&D × SFH*. Columns (3) and (4) report results of the robustness checks that include *Trend*^*2*^ and year fixed effects, respectively. The maximum variance inflation factor (VIF) was below 5 in the primary specification. It increased substantially when the quadratic time trend was added because of the strong correlation between *Trend* and *Trend*^*2*^.

**Table 3:** Pharmaceutical-industry R&D expenditure and the pharmaceutical cost ratio

	(1)	(2)	(3)	(4)
*Intercept*	−0.649***	−0.549**	0.416**	−0.021
	(−8.072)	(−2.862)	(2.583)	(−0.412)
*R&D*	0.059***	0.049**	−0.042**	
	(7.894)	(2.970)	(−2.839)	
*R&D × SFH*	0.066***	0.066***	0.066***	0.066***
	(3.968)	(3.928)	(3.888)	(3.459)
*Trend*		0.000	−0.003***	
		(0.461)	(−4.000)	
*Trend*^*2*^			0.001***	
			(6.728)	
Adj.R^2^	0.975	0.975	0.979	0.976
N	56	56	56	56
Category fixed effects	Included	Included	Included	Included
Year fixed effects	Excluded	Excluded	Excluded	Included
Max VIF	1.333	4.104	50.909	1.000

Notes: The table reports the results from hospital-category fixed-effects regressions. In parentheses, t-statistics with year-cluster robust standard errors are reported. ***, **, and * indicate statistical significance at the 1%, 5%, and 10% levels, respectively.

**Table 4:** Pharmaceutical-industry R&D expenditure and the medical operating margin

	(1)	(2)	(3)	(4)
*Intercept*	−0.538	0.790	−1.232	0.030
	(−1.438)	(0.930)	(−0.611)	(0.355)
*R&D*	0.053	−0.075	0.117	
	(1.308)	(−0.944)	(0.615)	
*R&D × SFH*	−0.086***	−0.086***	−0.086***	−0.086**
	(−3.155)	(−3.124)	(−3.092)	(−2.751)
*Trend*		0.006	0.012**	
		(1.244)	(2.511)	
*Trend*^*2*^			−0.001	
			(−1.229)	
Adj.R^2^	0.925	0.926	0.926	0.920
N	56	56	56	56
Category fixed effects	Included	Included	Included	Included
Year fixed effects	Excluded	Excluded	Excluded	Included
Max VIF	1.333	4.104	50.909	1.000

Notes: The table reports the results from hospital-category fixed-effects regressions. In parentheses, t-statistics with year-cluster robust standard errors are reported. ***, **, and * indicate statistical significance at the 1%, 5%, and 10% levels, respectively.

Table 3 shows that the coefficients on *R&D × SFH* were positive and statistically significant in all specifications. In Column (2), a one-standard-deviation increase in log R&D expenditure was associated with a 1.089 percentage point larger increase in the pharmaceutical cost ratio for the special functioning hospital category than for the other three categories (0.066 × 0.165), supporting H1.

By contrast, Table 4 indicates that the coefficients on *R&D × SFH* were negative and statistically significant in all specifications. The estimated association corresponding to a one-standard-deviation increase in log R&D expenditure was 1.419 percentage points more negative for the special functioning hospital category than for the other three categories (−0.086 × 0.165). This result is consistent with H2.

Because the sample contained only 14 fiscal-year clusters, wild cluster bootstrap *p*-values were additionally calculated for the coefficient on *R&D × SFH* (untabulated). The bootstrap *p*-value was 0.031 in each of the four pharmaceutical cost ratio specifications and 0.088 in each of the four medical operating margin specifications. Thus, the evidence for H1 remained statistically significant at the 5% level, whereas the evidence consistent with H2 remained statistically significant at the 10% level.

Taken together, these results indicate that the estimated associations between pharmaceutical-industry R&D expenditure and hospital financial performance were less favorable for the special functioning hospital category than for the other hospital categories. The coefficients on *R&D × SFH* retained the same signs and magnitudes across the alternative specifications. The wild cluster bootstrap results supported the statistical significance of the estimated associations, although the significance level differed between the two outcomes.

## 4 Discussion

### 4.1 Principal findings and strengths

This study found that the association between pharmaceutical-industry R&D expenditure and the pharmaceutical cost ratio was more positive for the special functioning hospital category than for the other categories. The association between pharmaceutical-industry R&D expenditure and the medical operating margin was also more negative for the special functioning hospital category than for the other categories. These findings remained consistent when a quadratic time trend was included and when the common linear trend was replaced with year fixed effects. Both associations also remained statistically significant under supplementary wild cluster bootstrap inference, at the 5% level for the pharmaceutical cost ratio and at the 10% level for the medical operating margin.

A key strength of this study is its integration of financial data for institutionally defined hospital categories with firm-level R&D data aggregated into an industry-level measure aligned with the hospital fiscal year. This approach enabled an examination of whether the associations between pharmaceutical-industry R&D expenditure and financial performance differed between the special functioning hospital category and the other hospital categories.

### 4.2 Contribution and implications

This study contributes to the literature on hospital financial performance. A previous systematic review identified environmental, structural, and operational factors as important determinants of hospital profitability and found that hospital size and occupancy rates were positively associated with profitability in many studies [27]. This study extends this literature by examining an external industry-level factor, pharmaceutical-industry R&D expenditure, and its associations with the pharmaceutical cost ratio and medical operating margin. It provides a novel perspective by linking pharmaceutical-industry R&D expenditure with hospital-category financial data and examining whether the estimated associations differ across hospital functions.

By combining hospital financial data with R&D information derived from pharmaceutical firms' financial reports, this study also contributes to the financial accounting literature on aggregate accounting information. Several financial accounting studies have aggregated accounting information from individual firms and investigated its usefulness [28], [29], [30], [31], [32], [33], [34], [35]. This research stream applies the informational value of accounting data to macro-level issues of academic and professional importance [36]. Previous studies have shown that aggregate earnings of listed firms contain information relevant to gross domestic product growth [29], [32], [33], [35], consumer prices [34], and monetary policy [30]. The current study extends this research stream by documenting associations between an industry-level R&D measure constructed from pharmaceutical firms' accounting data and hospital-category financial indicators.

The findings also have implications for discussions on hospital financing under regulated reimbursement and prospective payment systems. The stronger category-level associations observed for the special functioning hospital category are consistent with the possibility that financial pressures associated with newly developed and high-cost pharmaceuticals differ across hospital functions. This possibility warrants further investigation but does not, by itself, support specific financing policies.

### 4.3 Limitations and future research directions

This study has several limitations. First, this study used only Japanese data, which may limit the generalizability of the findings to countries with different pharmaceutical pricing and health systems. Given the global concern over rising drug costs, further research in other institutional settings is needed to determine whether similar associations are observed internationally.

Second, the analysis was based on a small sample of category-level observations. The limited number of categories and category-year observations restricts statistical precision and the range of covariates and alternative specifications that can be included. Hospital-category fixed effects account for time-invariant differences across categories. However, the aggregated data did not permit direct control for time-varying hospital-level factors, such as occupancy rates [27]. Moreover, the findings describe category-level associations and should not be generalized to every hospital within a category. Substantial heterogeneity may therefore remain among individual hospitals within each category.

Third, the available data do not identify the specific pharmaceuticals purchased or administered by hospitals. Pharmaceutical-industry R&D expenditure represents an input into a lengthy and uncertain drug-development process and is not a direct measure of new-drug output, drug prices, or hospital pharmaceutical use. Greater exposure of special functioning hospitals to innovative and high-cost medicines therefore represents one possible interpretation of the results rather than an identified mechanism. Consequently, this study cannot determine whether changes in the pharmaceutical cost ratio were associated with the use of newly developed pharmaceuticals or with changes in the volume or composition of existing pharmaceuticals. Although the proposed conceptual mechanism links pharmaceutical-industry R&D expenditure, drug development and pricing, hospital pharmaceutical costs, and hospital financial performance, the intermediate stages of this pathway were not directly observed. The analysis therefore does not identify a mediating pathway from pharmaceutical-industry R&D expenditure to hospital financial performance.

Fourth, the fixed-effects specification does not eliminate potential bias arising from time-varying unobserved factors. Because this study did not use random assignment, a natural experiment, or another source of exogenous variation, the estimated associations should not be interpreted as causal effects. Moreover, the findings do not establish that individual special functioning hospitals generally experience greater financial pressures associated with pharmaceutical-industry R&D expenditure.

Future research should combine individual hospital financial data with detailed information on pharmaceutical use. Hospital-level characteristics associated with financial distress, such as an indicator of unprofitable district hospitals [37], should also be considered. Such data would permit an assessment of within-category heterogeneity and clarify the intermediate pathways linking pharmaceutical-industry R&D expenditure to hospital financial performance. They would also provide a stronger evidentiary basis for evaluating alternative hospital funding policies.

## 5 Conclusion

This study examined the associations of pharmaceutical-industry R&D expenditure with the pharmaceutical cost ratio and medical operating margin, focusing on the special functioning hospital category in Japan. The association between pharmaceutical-industry R&D expenditure and the pharmaceutical cost ratio was more positive for the special functioning hospital category than for the other categories. The association between pharmaceutical-industry R&D expenditure and the medical operating margin was also more negative for this category than for the other categories.

These findings suggest that financial pressures related to pharmaceutical-industry R&D and high-cost medicines may differ across hospital functions. Greater exposure of special functioning hospitals to innovative and high-cost medicines represents one possible explanation, but this mechanism was not directly established because the development, pricing, purchase, and use of specific medicines were not observed. Moreover, because the analysis used aggregated category-level data and did not employ a causal research design, the findings do not establish causal effects for individual hospitals and do not directly justify targeted funding for special functioning hospitals. Future research using hospital-level data on pharmaceutical use and financial performance is needed to determine whether specific forms of targeted financial support would be warranted.

## Role of the funder

The funder had no role in the study design; data collection, analysis, or interpretation of data; writing of the report; or the decision to submit the article for publication.

## CRediT authorship contribution statement

**Yuto Yoshinaga:** Writing – review & editing, Writing – original draft, Validation, Methodology, Investigation, Funding acquisition, Formal analysis, Data curation, Conceptualization. **Takahiro Inoue:** Writing – review & editing, Writing – original draft, Data curation.

## Funding

This project is funded by the 10.13039/501100001691Japan Society for the Promotion of Science (Grant number 25K16768).

## Declaration of competing interest

The authors declare the following financial interests/personal relationships which may be considered as potential competing interests:

Yuto Yoshinaga reports that financial support was provided by Japan Society for the Promotion of Science. Takahiro Inoue declares that he has no known competing financial interests or personal relationships that could have appeared to influence the work reported in this paper.

## Data Availability

This study was supported by publicly accessible data in Survey on the actual state of medical economics (survey of medical institutions, etc.) (https://www.mhlw.go.jp/bunya/iryouhoken/database/zenpan/iryoukikan.html) and in firms' public disclosures as stated in the supplementary materials. It was also supported by third-party data in the NEEDS-Financial Quest (https://needs.nikkei.co.jp/services/financial-quest/) and eol (https://www.indb.co.jp/service/corporate_data/eol/).

## Appendix A Supplementary data

Supplementary material 1 Dataset.

Supplementary material 2 Manually collected R&D data.
